# Fucoidan Derived from *Fucus vesiculosus* Inhibits the Development of Human Ovarian Cancer via the Disturbance of Calcium Homeostasis, Endoplasmic Reticulum Stress, and Angiogenesis

**DOI:** 10.3390/md18010045

**Published:** 2020-01-09

**Authors:** Hyocheol Bae, Jin-Young Lee, Changwon Yang, Gwonhwa Song, Whasun Lim

**Affiliations:** 1 Department of Biotechnology, College of Life Sciences and Biotechnology, Korea University, Seoul 02841, Korea; bhc7@korea.ac.kr (H.B.); ycw117@korea.ac.kr (C.Y.); 2 Department of Pharmacology and Toxicology, Medical College of Wisconsin, Milwaukee, WI 53226, USA; jylee@mcw.edu; 3 Department of Food and Nutrition, College of Science and Technology, Kookmin University, Seoul 02707, Korea

**Keywords:** fucoidan, marine drug, ovarian cancer, angiogenesis, calcium homeostasis

## Abstract

Marine organisms are sources of several natural compounds with potential clinical use. However, only a few marine-based pharmaceuticals have been approved for use due to limited knowledge on their biological activities. Here, we identified the functional role of fucoidan extracted from *Fucus vesiculosus* on ovarian cancer. Fucoidan increased the death of ES-2 and OV-90 cells, through a reduction in proliferation, cell cycle arrest, releases of cytochrome c, reactive oxygen species (ROS) generation, and endoplasmic reticulum (ER) stress. Additionally, fucoidan increased the concentration of cytosolic and mitochondrial calcium in both cells. The decrease of cell proliferation was controlled by the inactivation of PI3K and MAPK signaling cascades in ES-2 and OV-90 cells. In a toxicity assay with normal zebrafish larvae, fucoidan did not induce toxicity, cardiotoxicity, development, kinesis, and apoptosis at different concentrations. However, it disrupted tumor formation and vascular development in a zebrafish xenograft model and angiogenesis transgenic (Tg, fli1-eGFP) model, respectively. Collectively, the results indicate that fucoidan may be a novel pharmaceutical for the management of human ovarian cancer.

## 1. Introduction

Ovarian cancer is the leading cause of death among gynecological diseases [1]. It is responsible for about 22,240 new occurrences and 14,070 deaths and it is the 5th cause of cancer-related deaths in the USA in 2018 [2]. The high mortality rate is due to the absence of early symptoms, late diagnosis, limited therapeutic approaches, and the emergence of drug resistance [3]. When ovarian cancer is caught early, the 5-year survival rate of patients is close to 90%, but in most cases, the cancer is detected after metastasis, leading to low 5-year survival rate (less than 30%) [4]. Additionally, it has a high recurrence rate of about 70% and is resistant to already used anticancer drugs [5]. Of all ovarian cancers, epithelial ovarian cancer (EOC) in the ovarian surface epithelium is the most prevalent. More than 75% of EOC patients are diagnosed after the cancer has spread to various parts of the peritoneum and the body [6]. At this advanced stage, there is low treatment efficacy, disease recurrence, and anticancer drug tolerance [7]. Thus, it is necessary to discover novel therapeutic substance to overcome chemoresistance and identify new chemotherapeutic agents for the treatment of EOC.

Fucoidan is an extract of various brown algae such as *Cladosiphon okamuranus*, *Fucus vesiculosus*, *Undaria pinnatifida*, and *Sargassum fulvellum* (Turner) C. Agardh. It has a complicated structure of sulfated polysaccharides [8,9]. Fucoidan has extensive physiological activities such as anti-coagulant [10], antioxidant [11], immuno-modulatory [12], anti-inflammatory [13], anti-bacterial activity [14], and anti-obesity [15,16] properties. Additionally, many studies suggest that fucoidan has an anti-cancer effect in various cancer cells such as leiomyosarcoma [17], bladder cancer [18], mastocarcinoma [19], colorectal cancer [20], and hepatocellular carcinoma [21]. There are several studies confirming the effects of fucoidan in ovarian cancer. Fucoidan reduces the density of ovarian cancer cells [22]. Fucoidan also reduces the number of viable ovarian cancer cells, depending on the type of cell line [23]. In addition, fucoidan from *Undaria pinnatifida* or *Fucus vesiculosus* reduces the development of ovarian cancer cells [24]. Fucoidan derived from *Fucus vesiculosus* has been shown to improve tamoxifen activity in ovarian cancer orthotopic mouse models [25]. However, there is a lack of understanding of the molecular system by which fucoidan inhibits the development of ovarian cancer cells. Therefore, here, we identified the inhibitory effects of fucoidan on the development of ovarian cancer in vitro and in vivo. We aimed to: (1) identify the efficacy of fucoidan on the alteration of cellular properties of ovarian cancer cells (ES-2 and OV-90 cells); (2) identify the fucoidan-mediated intracellular signaling pathways that affect the growth of ovarian cancer cells; (3) demonstrate the synergistic effects of fucoidan with cisplatin or paclitaxel against the progression and angiogenesis of cancer cells; and (4) determine the anti-cancer efficacy of fucoidan on ovarian cancer cells transplanted with zebrafish model. Collectively, we provide, to our knowledge, first report of fucoidan-induced apoptosis in ovarian cancer, indicating its possible use as an anti-cancer agent for the management of ovarian cancer progression.

## 2. Results

### 2.1. Fucoidan Regulates Proliferation and Apoptosis of Ovarian Cancer

**Figure 1:**
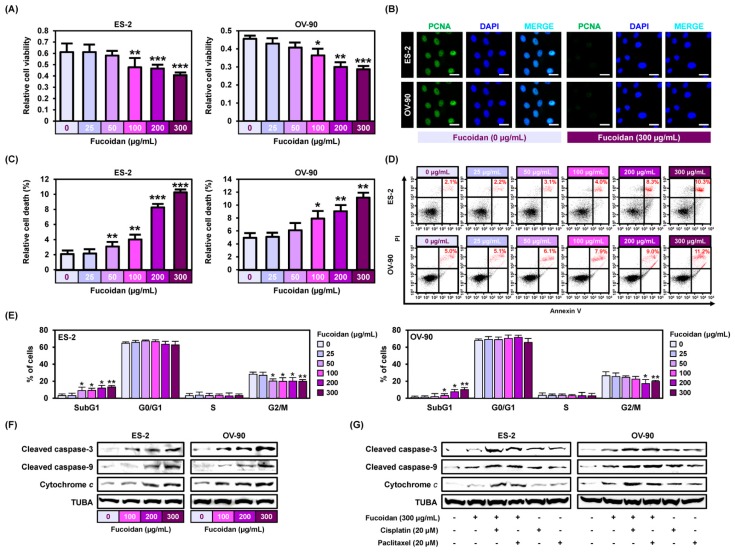
Fucoidan leads to cell death in ES-2 and OV-90 cells. (**A**) Observation of cell growth shows that fucoidan inhibits ES-2 and OV-90 cell growth. (**B**) Immunofluorescence observation of PCNA in ES-2 and OV-90 cell lines. Detection of PCNA in the nucleus of ES-2 and OV-90 cell lines was decreased by fucoidan. Scale bar indicates 40 µm. (**C**,**D**) Fucoidan-induced apoptosis of ES-2 and OV-90 cells. Fucoidan induced late apoptosis phase (red color) ES-2 and OV-90 cells concentration dependently (0, 25, 50, 100, 200, and 300 μg/mL) using a flow cytometry. (**E**) Population of the cells within each cell cycle progression was observed through flow cytometry after staining the fucoidan-treated ES-2 and OV-90 cells using PI dye. (**F**) Effects of fucoidan on the activation of apoptotic signals in ES-2 and OV-90 cells. (**G**) Effects of fucoidan with cisplatin or paclitaxel on the activation of the apoptotic proteins in human ovarian cancer cell lines. *** *p* < 0.001, ** *p* < 0.01, and * *p* < 0.05 indicate significant differences.

To identify the effect of fucoidan on the cell proliferation of ovarian cancer, we performed cell proliferation assays to observe DNA synthesis in ES-2 and OV-90 cells (Figure 1A). As the concentration of fucoidan increased (100–300 μg/mL), the cell growth inhibition increased. Immunoreactive proliferating cell nuclear antigen (PCNA) was mostly detected in the nucleus of vehicle-treated ES-2 and OV-90 cell lines (Figure 1B). However, the expression of PCNA dramatically inhibited in ES-2 and OV-90 cells by fucoidan (300 μg/mL) treatment. Additionally, the apoptotic ovarian cancer cells in response to fucoidan (0, 25, 50, 100, 200, and 300 μg/mL) were estimated using a flow cytometry following annexin V and PI assay (Figure 1C,D). In line with the decrease in cellular proliferation, fucoidan gradually increased cell death by 10.3% (*p* < 0.001) and 11.2% (*p* < 0.01) in ES-2 and OV-90 cell lines. Additionally, the sub-G1 phase of the cell cycle was significantly elevated in fucoidan-treated ES-2 and OV-90 cells, whereas S and G2/M phases were slightly decreased under the same condition (Figure 1E). In addition, fucoidan activated the cleaved caspase-3 and the cleaved caspase-9 and the release of cytochrome c in ES-2 and OV-90 cell lines (Figure 1F). The protein expression of cleaved caspases and cytochrome c was higher in ES-2 and OV-90 cells co-treated with fucoidan and chemotherapeutic agents (cisplatin or paclitaxel) than in the cells treated with fucoidan, cisplatin, or paclitaxel alone (Figure 1G).

### 2.2. Efficacy of Fucoidan on ROS Generation, Calcium Homeostasis, and Mitochondrial Membrane Potentials in Ovarian Cancer

**Figure 2:**
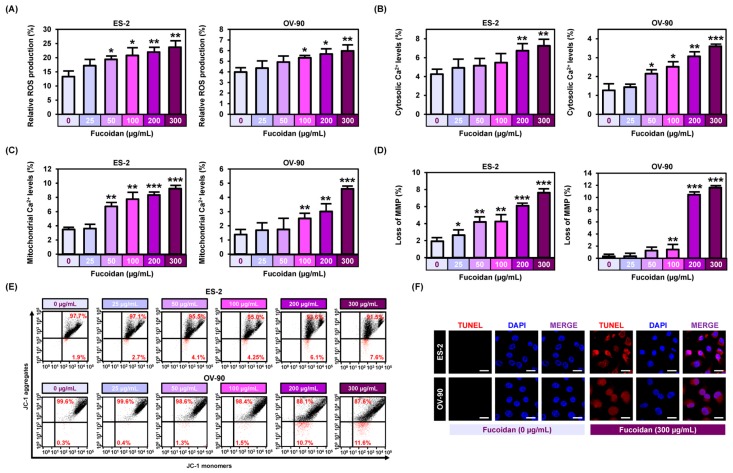
Induction of reactive oxygen species (ROS) production, calcium ion regulation, and mitochondrial membrane permeabilization by fucoidan. (**A**) ROS generation by fucoidan was estimated through dichlorofluorescein (DCF) fluorescence intensity using flow cytometry. (**B**) Flow cytometric observation of cytosolic Ca^2+^ in response to 48 h incubation with dose dependent fucoidan by staining ES-2 and OV-90 cells with Fluo-4. (**C**) Flow cytometric observation of mitochondrial Ca^2+^ concentrations in ES-2 and OV-90 cells in response to fucoidan by staining with Rhod-2. (**D**,**E**) Flow cytometric observation of mitochondrial membrane potential (MMP) in ES-2 and OV-90 cells treated with fucoidan by staining with JC-1 dye. Loss of MMP was estimated through JC-1 red and green fluorescence ratios. (**F**) Terminal deoxynucleotidyl transferase dNTP nick end labeling (TUNEL) detected apoptosis (red) and the cells were counter-stained with DAPI (blue) in ES-2 and OV-90 cells. Scale bar indicates 40 µm. *** *p* < 0.001, ** *p* < 0.01, and * *p* < 0.05 indicate significant difference.

**Figure 3:**
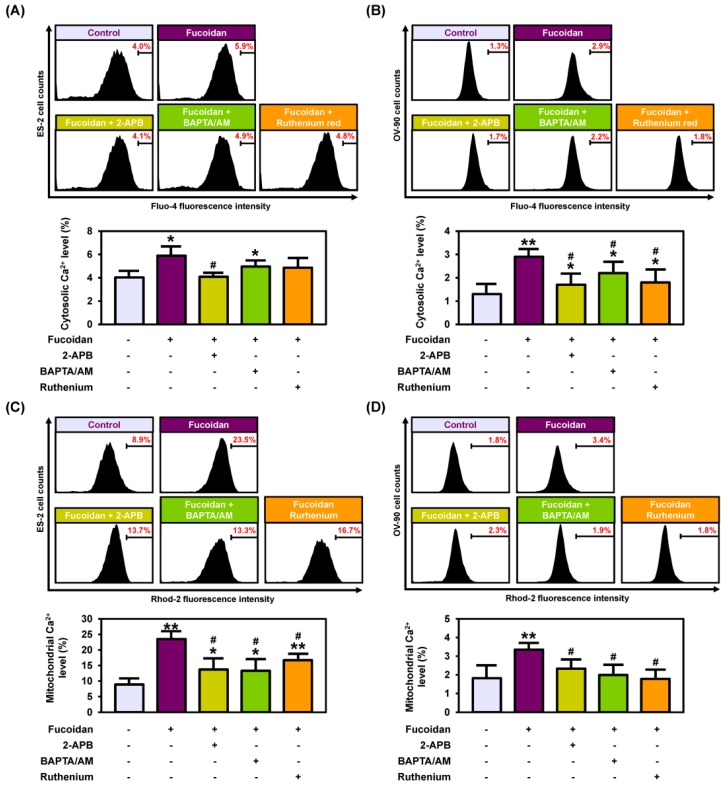
Effect of fucoidan with 2-APB, BAPTA/AM or ruthenium red on calcium concentration in the cytosol and mitochondria. (**A**,**B**) Flow cytometry of cytosolic Ca^2+^ with co-treatment of fucoidan with 2-APB, BAPTA/AM, or ruthenium red for 48 h in ES-2 and OV-90 cells. (**C**,**D**) Flow cytometry of mitochondrial Ca^2+^ concentrations in ES-2 and OV-90 cells by the combined treatment of fucoidan and 2-APB, BAPTA/AM or ruthenium red for 48 h. ** *p* < 0.01 and * *p* < 0.05 indicate significant effects as compared to the vehicle treated control. # indicates significant effects (*p* < 0.05) as compared to fucoidan alone.

To determine fucoidan-regulated cell properties of ovarian cancer, we analyzed reactive oxygen species (ROS) production using DCFH-DA analyzed by flow cytometry (Figure 2A). Fucoidan (0, 25, 50, 100, 200, and 300 μg/mL) gradually increased the production of ROS levels in both ES-2 and OV-90 cells up to 23.7% (*p* < 0.01) and 6.0% (*p* < 0.01). To identify alterations in cytosolic and mitochondrial calcium ion concentration, we stained the cells with Fluo-4 and Rhod-2 dyes (Figure 2B,C). In both ES-2 and OV-90 cells, 300 μg/mL fucoidan increased the cytosolic calcium ion levels by 7.3% (*p* < 0.01) and 3.6% (*p* < 0.001; Figure 2B). Similarly, the level of mitochondrial calcium ion rose by 9.2% (*p* < 0.001) and 4.6% (*p* < 0.001) in ES-2 and OV-90 cells, under the same conditions as above (Figure 2C). Furthermore, we stained ES-2 and OV-90 cells with JC-1 dye after treating with fucoidan at the indicated concentrations to analyze if the change in the levels of mitochondrial calcium ion was due to the disruption of MMP by fucoidan (Figure 2D,E). Treatment with 300 μg/mL fucoidan induced the loss of MMP by 7.6% (*p* < 0.001) in ES-2 and 11.6% (*p* < 0.001) in OV-90 cells. In addition, fucoidan treatment induced fragmentation of nuclear DNA in ES-2 and OV-90 cells, evidenced by an increased terminal deoxynucleotidyl transferase dNTP nick end labeling (TUNEL) reaction (Figure 2F). Next, we used three chemicals (2-APB, BAPTA/AM, and ruthenium red) to confirm if the cellular activity in response to fucoidan was directly mediated by calcium regulation. In both cells, the fucoidan activated intracellular calcium ion concentration was attenuated by all three chemicals (Figure 3A,B). Similarly, increased mitochondrial calcium ion levels induced by fucoidan were reduced by treatment with the combination of fucoidan with each calcium ion regulator (Figure 3C,D).

### 2.3. Fucoidan Regulates Intracellular Signals in Ovarian Cancer Cell Lines

**Figure 4:**
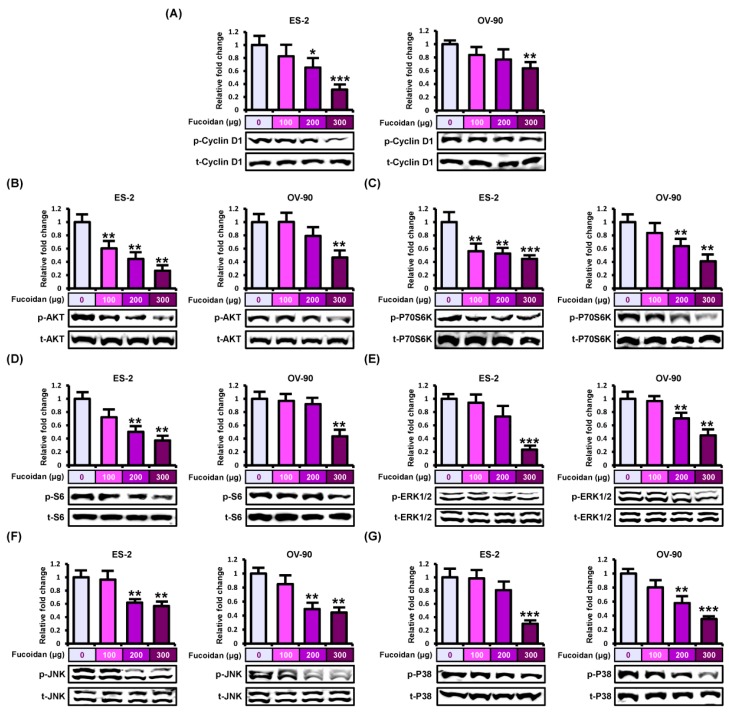
Repression of cyclin D1, PI3K, and MAPK pathways by fucoidan. (**A**–**G**) Western blotting indicated (**A**) phosphorylated (p)-cyclin D1, (**B**) p-AKT, (**C**) p-P70S6K, (**D**) p-S6, (**E**) p-ERK1/2, (**F**) p-JNK, and (**G**) p-P38 proteins in fucoidan (0, 100, 200, and 300  μg/mL)-treated ES-2 and OV-90 cells. The values of graph were calculated compared with vehicle-treated cells. *** *p* < 0.001, ** *p* < 0.01, and * *p* < 0.05 shows significances.

**Figure 5:**
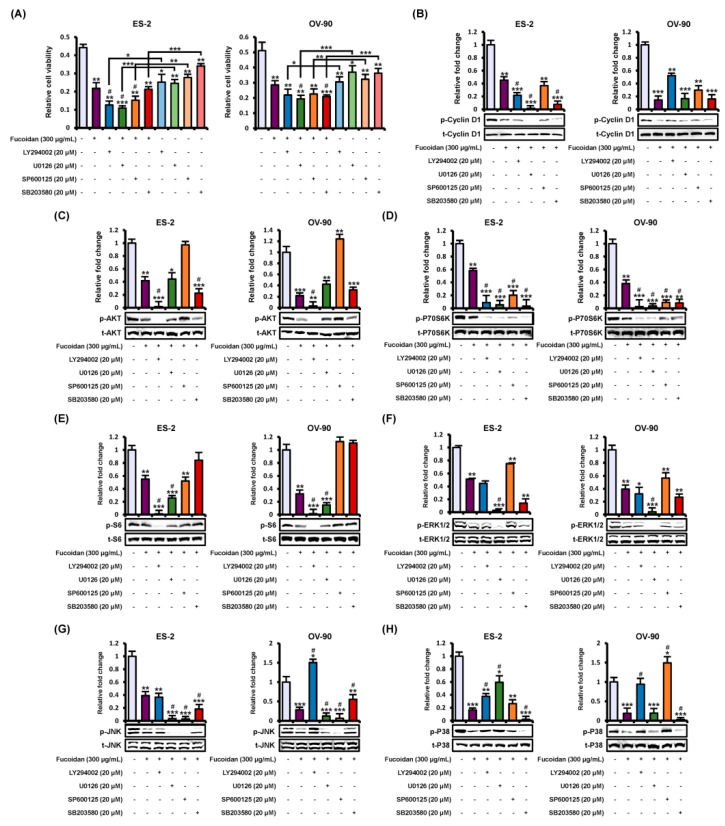
Inhibition of intracellular signals on cell growth and phosphorylation of target proteins in ovarian cancer cell. (**A**) The growth of ES-2 and OV-90 cells was observed by treatment of fucoidan (300 μg/mL) or a combination of fucoidan with LY294002 (blockage of AKT), U0126 (blockage of ERK1/2), SB203580 (blockage of P38), or SP600125 (blockage of JNK) for 48 h. (**B**–**H**) Repression of inhibitors on the activation of (**B**) p-cyclin D1, (**C**) p-AKT, (**D**) p-P70S6K, (**E**) p-S6, (**F**) p-ERK1/2, (**G**) p-JNK, and (**H**) p-P38 in both ES-2 and OV-90 cells. The values of graph were calculated compared with vehicle-treated cells. *** *p* < 0.001, ** *p* < 0.01, and * *p* < 0.05 indicate significant effects of each treatment compared with vehicle-treated control cells. # indicates significant effects (*p* < 0.05) as compared to fucoidan alone.

To elucidate the fucoidan-induced intracellular signal transduction associated with proliferation and apoptosis, we conducted western blotting to investigate the phosphorylation of protein kinases including PI3K and MAPK pathways (Figure 4). Phosphorylated cyclin D1, a key regulator of the cell cycle, was gradually inhibited by fucoidan (0, 100, 200, and 300 μg/mL) in ES-2 (up to 0.31-fold, *p* < 0.001) and OV-90 (up to 0.63-fold, *p* < 0.01) cells (Figure 4A). Additionally, fucoidan significantly inhibited the activation of PI3K pathway signaling cascades such as AKT, P70S6K, and S6 in ES-2 and OV-90 cells (Figure 4B–D). Similarly, phosphorylated ERK1/2, JNK, and P38 proteins were reduced in fucoidan-treated ES-2 and OV-90 cells (Figure 4E–G). Therefore, fucoidan inactivated PI3K/MAPK signals in ES-2 and OV-90 cell lines. Furthermore, we compared the proliferation of ES-2 and OV-90 cells between treatment of fucoidan alone (300 μg/mL) and fucoidan with each pharmacological inhibitor including LY294002, U0126, SP600125, and SB203580. LY294002, U0126, SP600125, and SB600125 decreased cell growth in ES-2 and OV-90 cells. Moreover, the combined treatment of fucoidan and each inhibitor showed a synergistic anti-proliferative effect compared with fucoidan or each inhibitor alone (Figure 5A). To confirm the activity of protein kinases, ovarian cancer cells pre-treated with the inhibitors before the incubation with fucoidan (300 μg/mL) for the identification of specific fucoidan-mediated signaling cascades (Figure 5B–H). The phosphorylation of cyclin D1 was further decreased by the inhibition of EKR1/2 and P38 pathways using U0126 and SB203580 in ES-2 cells (Figure 5B). The decreased phosphorylation of AKT, P70S6K, and S6 was almost inhibited by a combination of fucoidan with LY294002 (a PI3K inhibitor) in both ES-2 and OV-90 cells compared with fucoidan alone (Figure 5B–D). However, the phosphorylation of AKT proteins was more activated in ovarian cancer cells treated with co-incubation of fucoidan and SP600125 than fucoidan alone. The phosphorylation of S6 proteins was more activated in ES-2 and OV-90 cells co-treated with fucoidan and SB203580 than fucoidan alone. The decreased phosphorylation of ERK1/2 was blocked by U0126 pre-treatment in both ES-2 and OV-90 cells comparison with fucoidan alone (Figure 5F). Although the decrease in JNK phosphorylation induced by fucoidan was inhibited by all the inhibitors in ES-2 cells, it was blocked by U0126 or SP600125 in OV-90 cells (Figure 5G). In comparison with fucoidan treatment alone, fucoidan-inactivated P38 was inhibited by SB203580 in ES-2 and OV-90 cells incubated with the co-treatment of fucoidan and SB203580 (Figure 5H).

### 2.4. Disruptiopn of Endoplasmic Reticulum (ER) by Fucoidan in Human Ovarian Cancer Cell Lines

**Figure 6:**
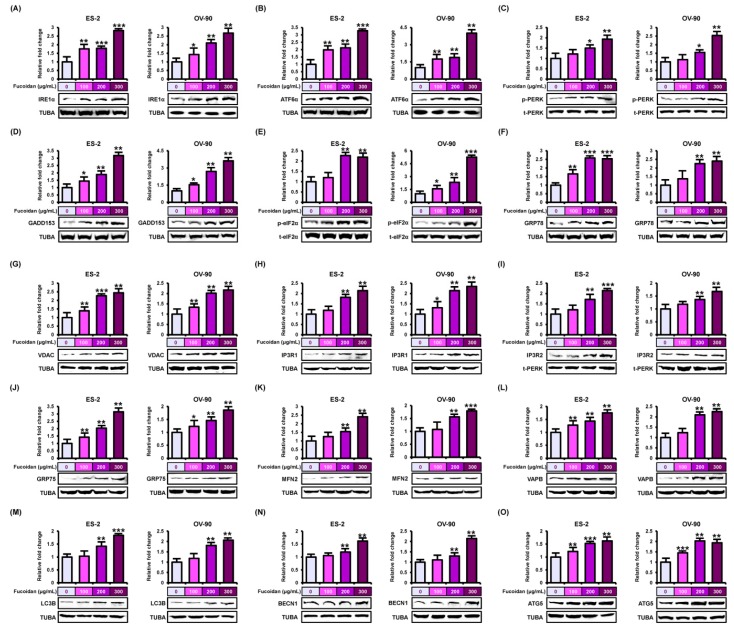
Stimulation of endoplasmic reticulum (ER) stress sensor, ER–mitochondria tethering proteins, and autophagy regulators by fucoidan in ES-2 and OV-90 cells. (**A**–**F**) The stimulation of ER stress sensor proteins, (**A**) IRE1α, (**B**) ATF6α, (**C**) p-PERK, (**D**) GADD153, (**E**) p-eIF2α, and (**F**) GRP78 was observed through western blot analysis in ES-2 and OV-90 cell lines incubated with fucoidan. (**G**–**L**) The activation of ER-mitochondria axis-associated proteins, (**G**) VDAC, (**H**) IP3R1, (**I**) IP3R2, (**J**) GRP75, (**K**) MFN2, and (**L**) VAPB was analyzed by western blotting in fucoidan treated ES-2 and OV-90 cells. (**M**–**O**) The activation of autophagy-associated proteins, (**M**) LC3B, (**N**) BECN1, and (**O**) ATG5 was observed through western blot in ES-2 and OV-90 cells incubated with fucoidan. The graph of the signals was calculated compared with total signal or α tubulin (TUBA). *** *p* < 0.001, ** *p* < 0.01, and * *p* < 0.05 show significances.

To discover the efficacy of fucoidan on endoplasmic reticulum (ER) stress in ES-2 and OV-90 cell lines, we performed western blotting using proteins derived from both cell lines incubated with fucoidan (0, 100, 200, and 300 μg/mL). In fucoidan-treated ES-2 and OV-90 cell lines, the unfolded protein response (UPR)-signals (ER stress inducers)—inositol-requiring enzyme 1α (IRE1α), activating transcription factor 6α (ATF6α), and PKR-like ER resident kinase (PERK)—were activated in the ER transmembrane (Figure 6A–C). Additionally, we confirmed that the cascades of the ER stress sensor-growth arrest- and DNA damage-inducible gene 153 (GADD153) and phosphorylated eukaryotic translation-initiation factor 2a (p-eIF2a) increased in fucoidan-treated ovarian cancer cells (Figure 6D,E). Moreover, the upstream of ER stress sensor—glucose-regulated protein 78 (GRP78) gradually increased in ovarian cancer cells by the increase in the concentration of fucoidan (Figure 6F). Next, after fucoidan treatment on ES-2 and OV-90 cells, we confirmed the expression of proteins related to the ER–mitochondria axis (Figure 6G–L). Protein expression of voltage-dependent anion channel (VDAC), inositol 1,4,5-triphosphate receptor 1 (IP3R1), IP3R2, glucose-regulated protein 75 (GRP75), mitofusin 2 (MFN2), and vesicle-associated membrane protein-associated protein B/C (VAPB) all increased in ES-2 and OV-90 cells following fucoidan treatment. In addition, protein expression of microtubule-associated proteins 1A/1B light chain 3B (LC3B), beclin-1 (BECN1), and autophagy related 5 (ATG5), proteins involved in autophagy, also increased in response to fucoidan treatment (Figure 6M–O).

### 2.5. In Vivo Toxicity and Xenograft Analysis of Fucoidan Using Zebrafish

**Figure 7:**
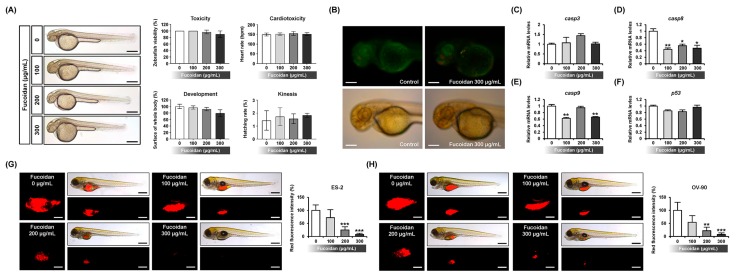
Effects of fucoidan on normal and xenograft zebrafish in vivo. (**A**) Toxicological analysis of fucoidan (0, 100, 200, and 300 μg/mL) was performed using fucoidan-exposed zebrafish larvae for 48 h to evaluate toxicity, cardiotoxicity, development, and kinesis in vivo. Scale bar indicates 250 µm. (**B**) Apoptotic effects of fucoidan on zebrafish larvae was analyzed using acridine orange to detect apoptotic cells after 48 h incubation. (**C**–**F**) Expression of apoptosis-related genes was estimated in fucoidan-treated zebrafish larvae for 48 h by quantitative RT-PCR. The scale bar indicates 50 µm. (**G**,**F**) In zebrafish xenograft model generated with microinjection of ES-2 (**G**) and OV-90 (**H**) cells, pre-treated fucoidan gradually inhibited tumor formation leading to a decrease in tumor size in vivo. The scale bar reveals 25 μm in square panels and 100 μm in rectangle panels. *** *p* < 0.001, ** *p* < 0.01, and * *p* < 0.05 indicate significant effects of fucoidan.

To validate the toxic effect of fucoidan, we performed toxicity assay of the response of normal zebrafish embryos to fucoidan (Figure 7A). Fucoidan did not induce toxicity, cardiotoxicity, and kinesis in the zebrafish embryos. Although their development was slightly decreased, there was no significant efficacy of fucoidan on the development of the embryos. To determine the effects of fucoidan on apoptosis in vivo, the zebrafish embryos were incubated with fucoidan and the apoptotic cells were tagged with acridine orange (Figure 7B). Our results showed that the apoptotic cells indicated by green fluorescence within the embryos were rarely detected in fucoidan-treated zebrafish. Although the expression of apoptosis-related genes including *casp8* and *casp9* decreased in 300 μg/mL fucoidan-treated zebrafish as indicated by quantitative RT-PCR analysis, there was no difference in the expression of *casp3* and *p53* genes in comparison with the control (Figure 7C–F). There was an efficient suppression of tumor volume and formation in fucoidan-treated transgenic model compared with the vehicle-treated model (Figure 7G,H).

### 2.6. Inhibitory Effects of Fucoidan on Angiogenesis In Vivo and In Vitro

**Figure 8:**
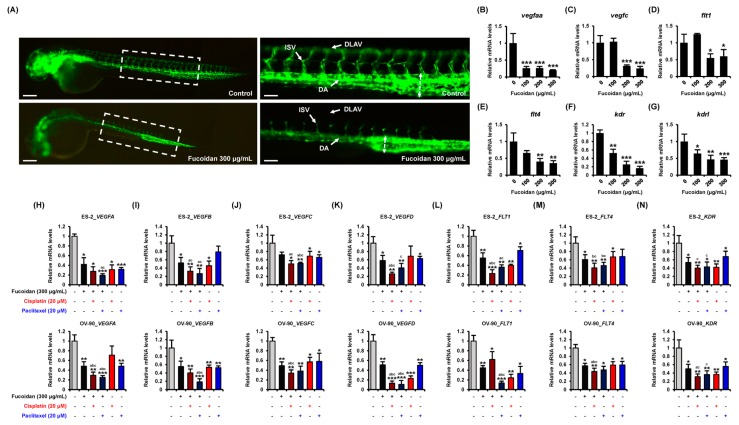
Anti-angiogenic activity of fucoidan in vivo and in vitro. (**A**) Zebrafish fli1 Tg (eGFP) models were analyzed to visualize vascular development in response to fucoidan. Fucoidan disrupted vasculature development, especially dorsal longitudinal anastomotic vessel (DLAV), dorsal aorta (DA), and intersegmental vessels (ISVs). Scale bar indicates 300 μm in left panels and 60 μm in right magnified panels. (**B**–**G**) Expression of angiogenesis-related genes was validated in fucoidan-treated fli1 Tg zebrafish by quantitative RT-PCR analysis. (**H**–**N**) Reduction of angiogenesis-related genes in ES-2 and OV-90 cells with the combination of fucoidan and cisplatin or paclitaxel. Messenger RNA expression of target genes was calculated based on that of *GAPDH* gene. *** *p* < 0.001, ** *p* < 0.01, and * *p* < 0.05 indicate significant effects of each treatment compared with vehicle-treated control cells. # represents significances (*p* < 0.05) compared with fucoidan alone.

To identify if fucoidan has anti-angiogenic effects, we used the zebrafish Tg(fli1:eGFP) line generated for the study of vascular system. Treatment with fucoidan (300 μg/mL) disrupted the vascular development of zebrafish embryo, especially dorsal longitudinal anastomotic vessel (DLAV), intersegmental vessel (ISV), and dorsal aorta (DA) parts compared with vehicle-treated fli1 Tg models (Figure 8A). In accordance with the results, the angiogenesis-related genes such as *vascular endothelial growth factor Aa* (*vegfaa*), *vegfc*, *fms related tyrosine kinase 1* (*flt1*), *flt4*, *kinase insert domain receptor* (*kdr*), and *kdr like* (*kdrl*) were dramatically decreased in fucoidan-incubated fli1 Tg zebrafish compared with the control (Figure 8B–G). Next, we compared the anti-angiogenic effects of fucoidan with conventional anti-cancer drugs including cisplatin and paclitaxel in human ovarian cancer cell lines. The mRNA expression of *VEGFs* (*VEGFA*-*VEGFD*) was reduced by fucoidan in ES-2 and OV-90 cells (Figure 8H–K). Their expression was synergistically decreased by a combination of fucoidan with cisplatin or paclitaxel compared with individual treatment. Even though the synergy of fucoidan with cisplatin or paclitaxel on *FLT1* expression was indicated only in ES-2 cells, mRNA expression of *FLT4* and *KDR* was highly inhibited in ES-2 and OV-90 cells incubated with the combined treatment compared with those incubated with the individual treatments (Figure 8L–N).

## 3. Discussion

**Figure 9:**
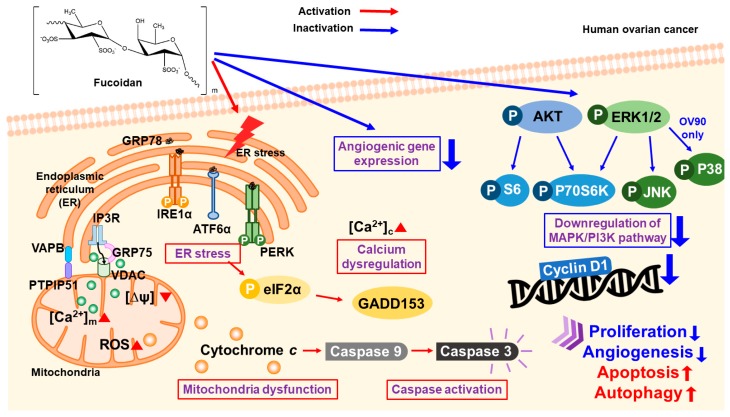
Illustration of fucoidan-regulated cell apoptosis in human ovarian cancer.

Fucoidan caused a 40% growth inhibition in ES-2 and OV-90 cells at a dose of 300 µg/mL, and it induced the apoptosis in ovarian cancer cells after 48 h incubation. Additionally, fucoidan triggered the depolarization of MMP, production of ROS, and an increase in calcium ion concentration in cytosol and mitochondria. Fucoidan inhibited PI3K/MAPK intracellular signal pathways; however, it activated the apoptotic cascades and ER stress sensor proteins in the ovarian cancer cells as illustrated in Figure 9. Although fucoidan did not affect the normal zebrafish in vivo, it highly decreased tumor formation and angiogenesis in the xenograft and fli1 Tg models, respectively. Additionally, fucoidan suppressed the expression of angiogenesis-related genes in vivo and in vitro by enhancing the efficiency of chemotherapeutic agents. These data indicate that fucoidan can be used as a novel drug for the management and treatment of ovarian cancer.

In recent decades, reports on the establishment of anti-cancer agents for the reduction of cancer-related death rates have increased tremendously. Since the side effects of synthetic agents and recurrence rate of ovarian cancer, the finding of anti-cancer agents from natural compounds is needed. Additionally, combination therapies of synthetic drugs and natural compounds have been studied for the improvement of chemosensitivity in ovarian cancer. Fucoidan, a macromolecule derived from brown algae, has been extensively studied due to their diverse biological functioning including anti-cancer, anti-oxidant, and anti-inflammatory effects. Fucoidan belongs to complex sulfated polysaccharides commonly existed in the cell walls of brown algae [14]. Although fucoidan has functional roles in the prevention of various cancers, its biological activities have not been reported in ovarian cancer.

In previous studies, fucoidan induced apoptotic mechanism in prostate cancer cells [26] and animal models [27]. Additionally, fucoidan stimulated cell death in bladder cancer [28] and colon cancer [18]. In agreement with these results, fucoidan inhibited cell growth and induced cell death in both ovarian cancer cells in our study. PCNA, a well-known marker for cell proliferation [29], was decreased by fucoidan in ES-2 and OV-90 cells. Similar to cell cycle progression regulated by fucoidan in several cancer cells including bladder cancer, colon cancer, and leukemia [30], fucoidan increased sub G1 phase and deceased G2/M phase in ES-2 and OV-90 cells. According to a previous study, fucoidan increased the generation of ROS in human ovarian cancer cell lines [31]. Previous reports suggested that fucoidan increased ROS generation, mitochondrial oxidative damage, MMP depolarization, production of cytochrome c, decrease of X-linked inhibitor of apoptosis protein (XIAP), and cleavage of caspase-3 and caspase-9, which are similar with our results [32].

Mitochondria are crucial organelles associated with the intrinsic apoptotic pathways [33]. Mitochondrial cell death is accompanied with activation of caspases, secretion of cytochrome *c*, increased mitochondrial Ca^2+^, mitochondrial membrane permeabilization and stimulation of pro-apoptotic signals such as Bax and Bak, [34]. The excess of mitochondrial Ca^2+^ can induce the secretion of pro-apoptotic signals through the destruction of the mitochondria. Additionally, malfunction of mitochondria in cancer cells promotes the alteration in the Ca^2+^ levels of cytosol and cell death [35]. Moreover, the intracellular free Ca^2+^ inhibits anti-apoptotic protein (Bcl-2) and activates pro-apoptotic proteins (Bad and Bcl-XL), leading to early or late apoptosis [36]. In our experiments, we used 2-APB, BAPTA/AM, and ruthenium red to understand the detailed calcium regulation of fucoidan. 2-APB inhibits calcium concentration through regulation of both IP3Rs and TRP channels [37,38]. Since BAPTA/AM has four carboxylic acid functional groups, it combines with two calcium ions and acts as a calcium chelator [39,40]. Ruthenium red is a potent inhibitor of intracellular calcium release by regulation of ryanodine receptors [41]. Fucoidan increased calcium concentration of ovarian cancer cells through IP3Rs, TRP channels and ryanodine receptors via ER-mitochondria tethering. Therefore, our study demonstrated that fucoidan induced the cell death of human ovarian cancer cells by increasing the concentration of calcium in both cytoplasm and mitochondria. Additionally, fucoidan stimulated the loss of MMP in ovarian cancer cell lines through the excess ROS levels.

Ca^2+^ and ROS can affect each other for maintaining cell physiology. ROS can regulate Ca^2+^ signals, and Ca^2+^ plays a vital role in ROS production. The increased Ca^2+^ concentration induces the activation of ROS producing enzymes and generation of free radicals [42]. High concentration of Ca^2+^ increases the ROS production through stimulation of respiratory chain activity. Moreover, the increased ROS induces Ca^2+^ releasing, ER stress, and further ROS production through ER-calcium channels. Therefore, the elevated ROS production and Ca^2+^ concentration can lead to mitochondrial swelling and cell death through induction of mitochondrial membrane permeability transition and releasing of pro-apoptotic factors [43].

The intracellular signals are crucial for the regulation of cell survival and proliferation. Fucoidan increased cell death in several cancers by inhibiting the activation of cyclin D1, cyclin E, cyclin-dependent-kinases (Cdks), PI3K, and MAPK [44,45,46]. Therefore, the inhibition of PI3K/AKT signals in cancer cells might be a useful method to the management of cancer [47]. Herein, we identified that fucoidan reduced the activities of cyclin D1, PI3K/MAPK signals. In other studies, fucoidan decreased the expression of anti-apoptotic signals including Bcl-2, Bcl-XL, and MCL-1 in breast cancer [48,49]. Furthermore, fucoidan promotes cell death in human hepatocellular carcinoma (HCC) cells through cleavage of caspases [50]. Moreover, fucoidan induces the death of HCC cells through activation of caspases-7, -8, and -9 [44]. Fucoidan upregulates toll-like receptor 4 (TLR4)/CHOP-mediated caspase-3 and poly (ADP-ribose) polymerase (PARP) stimulation to promote anti-cancer effects of cisplatin in human lung cancer cells [51]. Our results indicate that fucoidan increased cell death via the cleavage of caspase-3 and caspase-9 and the release of cytochrome c in ES-2 and OV-90 cells. Additionally, fucoidan showed synergistic effects with cisplatin or paclitaxel, causing ovarian cancer cell death. Moreover, ER play pivotal role in diverse intracellular mechanism including protein translocation, protein folding, and post-transcriptional modification [52]. Previous studies reported that fucoidan promotes cancer cell apoptosis through the induction of ER stress. Fucoidan induces cell death by regulating GRP78 and ER protein 29 in breast cancer and colorectal cancer [53]. Consistent with the result of these studies, fucoidan increased ER stress sensor signals such as IRE1α, ATF6α, PERK, GADD153, eIF2α, and GRP78 in ES-2 and OV-90 cells. Alternative pattern of signals associated with the ER-mitochondria axis regulated by fucoidan suggest that anti-cancer effect of fucoidan is accompanied with the interaction of ER and mitochondria. Angiogenesis is the development of new blood vessels and, here, fucoidan inhibited the angiogenesis of cancer cells. The angiogenesis network is crucial for provision of oxygen and nutrition in cancer cells [54]. Thus, the repression of angiogenesis is used in diverse anti-cancer therapies. Fucoidan decrease hypoxia-stimulated H_2_O_2_ production, hypoxia-inducible factor-1 (HIF-1) formation, generation of VEGF, cell migration, and invasion of T24 cells. Additionally, low molecular weight fucoidan inhibits tube generation through hypoxic HUVECs and blood capillary production in the cancer cells [55]. Fucoidan suppresses cancer metastasis through reducing of VEGF and matrix metalloproteinases in Lewis tumor-xenograft mice [56]. Similarly, treatment with fucoidan repressed the mRNA expression of angiogenesis related genes including *VEGFA*, *VEGFB*, *VEGFC*, *VEGFD*, *FLT-1*, *FLT-4*, and *KDR* in the ovarian cancer cells. Moreover, in in vivo angiogenic Tg zebrafish model, fucoidan completely suppressed vasculature development, whereas it did not affect normal zebrafish embryos. Collectively, fucoidan inhibited PI3K, MAPK, the mitochondrial-mediated apoptotic signaling, and angiogenesis and activated ER stress to induce cell death of human ovarian cancer cells.

## 4. Material and Methods

### 4.1. Chemicals

Fucoidan (catalog number: F8315), cisplatin (catalog number: P4394), and paclitaxel (catalog number: T7408) were bought from Sigma-Aldrich (St. Louis, MO, USA). The antibodies used are indicated in Table 1. U0126 (catalog number: EI282), SP600125 (catalog number: EI305), and SB203580 (catalog number: EI286) were bought from Enzo Life Sciences, Inc. (Farmingdale, NY, USA), and LY294002 (catalog number: 9901) was bought from Cell Signaling Technology, Inc. (Danvers, MA, USA).

### 4.2. Cell Culture

ES-2 and OV-90 were bought from American Type Culture Collection (Manassas, VA, USA) and incubated in McCoy’s 5A Medium (10% fetal bovine serum; FBS: heat inactivation before use; 56 °C, 30 min) at 37 °C in a CO_2_ incubator. The ovarian cancer cell lines were incubated with diverse doses of fucoidan with or not with inhibitors of various cell signals.

### 4.3. Cell Proliferation Analysis

Cell growth was observed through the Cell Proliferation ELISA, BrdU Kit (Roche, Basel, Switzerland) according to the manufacturer’s recommendations. ES-2 and OV-90 cell lines were incubated in a 96-well plate for 24 h in serum-free McCoy’s 5A Medium. Next, the cells were incubated with fucoidan or fucoidan with each signaling inhibitor such as LY294002, U0126, SP600125, and SB203580. After 48 h treatment, 10 mM BrdU was co-treated for 2 h at 37 °C. Next, the cells were fixed and treated with anti-BrdU-peroxidase working solution for 90 min. Lastly, the absorbance values of the cells with 3,3′,5,5′-tetramethylbenzidine were estimated at 370 nm and 492 nm using Epoch microplate spectrophotometer (BioTek, Winooski, VT, USA). The experiment was conducted triplicate.

### 4.4. Observation of Immunofluorescence

PCNA were observed using immunofluorescence microscopy. The cells were incubated with or without fucoidan (300 µg/mL) at 37 °C for 48 h in a CO_2_ incubator and tagged with mouse anti-human monoclonal PCNA antibody (Santa Cruz Biotechnology, Dallas, TX, USA). Next, cells were treated with goat anti-mouse IgG Alexa 488 (Invitrogen, Carlsbad, CA, USA) at a dilution of 1:200 for 1 h at room temperature. Then, the cells were rinsed through 0.1% BSA -PBS and double staining with 4′,6-diamidino-2-phenylindole (DAPI, Sigma-Aldrich, St. Louis, MO, USA). The image was observed via an LSM710 confocal microscope (Carl Zeiss, Oberkochen, Germany).

### 4.5. Annexin V and PI Staining

The death of ovarian cancer cell lines promoted by fucoidan was estimated through an FITC Annexin V apoptosis detection kit I (BD Biosciences, Franklin Lakes, NJ, USA). The ovarian cancer cell lines (5 × 10^5^ cells) were incubated on 6-well plates and treated with fucoidan (0, 25, 50, 100, 200, and 300 μg/mL) for 48 h at 37 °C. The cells were rinsed with PBS. The cell suspension (100 µL, 1 × 10^6^ cells) was stained with Annexin V (5 μL) and propidium iodide (PI; 5 μL) for 15 min at room temperature in the dark. The fluorescent intensity was determined through Guava easyCyte™ 5 Flow Cytometer (Merck Millipore, Burlington, MA, USA). The experiment was repeated three times.

### 4.6. Cell Cycle Assay

The cell cycle progression of ES-2 and OV-90 cells was observed through PI. The cells on 6-well plates were treated with fucoidan (0, 25, 50, 100, 200, and 300 μg/mL) for 48 h at 37 °C and 5% CO_2_. The cells were rinsed with PBS. Next, cells were resuspended (1 × 10^6^ cells) and treated with RNase A (5 μL) and PI (5 μL) for 30 min at room temperature in the dark. The fluorescent intensity was calculated through Guava easyCyte™ 5 Flow Cytometer (Merck Millipore, Burlington, MA, USA). The experiment was repeated three times.

### 4.7. Determination of Cellular ROS

Generation of ROS was observed by 2′,7′-Dichlorofluorescin diacetate (DCFH-DA, Sigma). The cells were rinsed with PBS, and stained with 10 µM DCFH-DA for 30 min at 37 °C. The cells were washed twice with PBS and incubated with diverse doses of fucoidan for 1 h at 37 °C and 5% CO_2_ condition. The cells were rinsed with PBS. The fluorescent DCF intensity was observed through Guava easyCyte™ 5 Flow Cytometer (Merck Millipore, Burlington, MA, USA). The experiment was performed triplicate.

### 4.8. Intracellular Level of Free Ca^2+^

ES-2 and OV-90 cell lines were incubated on 6-well plates for 24 h in no-FBS medium. The cells were incubated with fucoidan (0, 25, 50, 100, 200, and 300 μg/mL) or co-treatment of fucoidan (300 μg/mL) and calcium chelators including 2-aminoethoxydiphenyl borate (2-APB; D9754, Sigma-Aldrich, St. Louis, MO, USA), 1,2-bis(2-aminophenoxy)ethane-N,N,N′,N′-tetraacetic acid tetrakis (acetoxymethyl ester; BAPTA/AM; sc-202488, Santa Cruz Biotechnology, Dallas, TX, USA), or ruthenium red for 48 h at 37 °C and 5% CO_2_. The cells were collected through 0.25% trypsin-EDTA and washed through medium before staining with 3 μM Fluo-4 acetoxymethyl (AM) ester (Invitrogen, Carlsbad, CA, USA) at 37 °C and 5% CO_2_ for 20 min. Fluorescent intensity was observed through Guava easyCyte™ 5 Flow Cytometer (Merck Millipore, Burlington, MA, USA). The experiment was repeated three times.

### 4.9. Measurement of Mitochondrial Ca^2+^ Concentration

ES-2 and OV-90 cell lines were incubated on 6-well plates 24 h in no-FBS medium. The cells were then incubated with fucoidan (0, 25, 50, 100, 200, and 300 μg/mL) or a combination of fucoidan (300 μg/mL) with 2 μM 2-APB, 16 μM BAPTA/AM or 2 μM ruthenium red for 48 h at 37 °C and 5% CO_2_. The cells were collected through 0.25% trypsin-EDTA and washed through medium before staining with 3 μM Rhod-2 and incubated at 4 °C for 30 min. Fluorescence was observed through Guava easyCyte™ 5 Flow Cytometer (Merck Millipore, Burlington, MA, USA). The experiment was repeated three times.

### 4.10. Observation of Mitochondrial Membrane Potential

Mitochondrial membrane potential (MMP) were determined through a mitochondria staining kit (Sigma-Aldrich, St. Louis, MO, USA). The ovarian cancer cell lines were incubated with fucoidan (0, 25, 50, 100, 200, and 300 μg/mL) for 48 h at 37 °C and 5% CO_2_. The cells were stained with 200 × JC-1 at 37 °C and 5% CO_2_ for 20 min. The cells were rinsed before observation. Fluorescence was observed through Guava easyCyte™ 5 Flow Cytometer (Merck Millipore, Burlington, MA, USA). The experiment was conducted triplicate.

### 4.11. TUNEL Assay

ES-2 and OV-90 cell lines were incubated on confocal dish and then starved for 24 h in no-FBS media. Then fucoidan was treated at 37 °C and 5% CO_2_ for 48 h. After treatment, the ovarian cancer cells were fixed with 4% paraformaldehyde-PBS for 1 h. The ovarian cancer cells were washed with PBS and permeabilized with 0.1% Triton X-100 in 0.1% sodium citrate for 2 min 4 °C. TUNEL (terminal deoxynucleotidyl transferase dNTP nick end labeling) In Situ Cell Death Detection kit, TMR red (Roche, Basel, Switzerland) was used for staining for 1 h at 37 °C in the dark. The ovarian cancer cells were rinsed with PBS and double stained with DAPI (Sigma-Aldrich, St. Louis, MO, USA). Fluorescence intensity was detected through a LSM710 (Carl Zeiss, Oberkochen, Germany) confocal microscope.

### 4.12. Western Blot Analysis

The ovarian cancer cell lines were rinsed twice with chilled PBS (Ca^2+^ and Ma^2+^), then scrapped off the ovarian cancer cells with lysis buffer, the lysates were incubated for 30 min on ice. The supernatant was separated by centrifugation at 21,000× *g*, 4 °C for 15 min. The protein concentrations were measured by a Bradford assay (Bio-Rad, Hercules, CA, USA). Then we quantified the protein 20 μg and made equal volume of loading mixture. Denatured proteins were separated by SDS-PAGE, and transferred to nitrocellulose membranes. Membranes were observed through chemiluminescence detection (SuperSignal West Pico, Pierce, Rockford, IL, USA) and calculated by ChemiDoc EQ system and Quantity One software (Bio-Rad, Hercules, CA, USA). Total proteins and α-tubulin (TUBA) were used to calculate the activation of target signals.

### 4.13. In Vivo Toxicity and Xenograft Analysis

Wild-type *Danio rerio* were bred according to Korea University guidelines at 28.5 °C with 10 h/14 h dark/light cycles. To evaluate the toxicity of fucoidan, zebrafish embryos were obtained from natural spawning between one male and two female adult fish. The embryos were transferred to 24-well plates (*n* = 10, per well) under each condition and cultured for 24 h in 0.3% Danieau’s buffer (1740 mM NaCl, 21 mM KCl, 12 mM MgSO_4_·7H_2_O, 18 mM Ca(NO_3_)_2_, and 150 mM HEPES), and the fucoidan-exposed zebrafish larvae were evaluated for an additional 48 h. Viability, heartbeat, and malformation were evaluated as toxicity indicators. To establish a zebrafish xenograft model, ES-2 and OV-90 cell lines were first incubated with fucoidan for 22 h. Then, the cells were incubated for 2 h with CM-Dil dye (4 μM, Invitrogen, Carlsbad, CA, USA) as a cell tracker. Both cells (1 × 10^2^ cells) were injected into the yolk sac of zebrafish anesthetized with 0.02% tricaine (Sigma-Aldrich, St. Louis, MO, USA) 48 h post fertilization through a PV820 microinjector (World Precision Instruments, Sarasota, FL, USA). After the incubation of the zebrafish at 28.5 °C for 72 h in 24-well plates, the fluorescence of the zebrafish was measured using a fluorescence microscope (DM3000, Leica, Wetzlar, Germany). Red fluorescent tumors were estimated through ImageJ software (U.S. National Institute of Health, Bethesda, MD, USA).

### 4.14. In Vivo Apoptosis Analysis

The cell apoptosis in the zebrafish was determined using acridine orange staining. Zebrafish (*n* = 10) were incubated to each concentration of fucoidan for 48 h and were transferred to 5 µg/mL of Danieau’s buffer containing acridine orange followed by incubation in the dark for 1 h. After staining with acridine orange, the zebrafish embryos were rinsed twice with Danieau’s buffer and visualized using a fluorescence microscope (DM3000, Leica, Wetzlar, Germany).

### 4.15. Analysis of Angiogenesis in Transgenic Zebrafish

The larvae of the transgenic zebrafish were incubated with the fucoidan for 48 h, and GFP expression patterns of 10 zebrafish larvae per condition were evaluated. Images were observed using a fluorescence microscope (DM3000, Leica, Wetzlar, Germany).

### 4.16. RNA Isolation

Total RNA was extracted through TRIzol reagent (Invitrogen, Carlsbad, CA, USA). Total RNA was confirmed by agarose gel electrophoresis to identify RNA quality.

### 4.17. Quantitative PCR Analysis

All primers as illustrated in Table 2 were produced by Bioneer (Daejeon, Korea) according to GenBank database using Primer 3 (ver. 4.0.0). Quantitative PCR was conducted using SYBR Green (Sigma-Aldrich, St. Louis, MO, USA) and a StepOnePlus Real-Time PCR System (Applied Biosystems, Waltham, MA, USA). Gene expression was estimated by C_T_ value (cycle number). Compared gene expression was calculated by the 2^−ΔΔCT^ method. The glyceraldehyde-3-phosphate dehydrogenase (*GAPDH*) gene in human and zebrafish was used as the normalization of gene expression.

### 4.18. Significances

Quantitative analysis was identified through (ANOVA). All experiments were performed triplicate. All analysis of significance was identified through the error terms. A *p*-value ≤ 0.05 indicated statistical significance. Data is expressed as least-square means (LSMs) with standard errors.

## 5. Conclusions

Collectively, we provided the first evidence, to our knowledge, of fucoidan-mediated cell apoptosis in human ovarian cancer cells and in zebrafish models. Fucoidan induced the depolarization of MMP and the production of ROS, leading to the overload of calcium concentration in cytosol and mitochondria in both ES-2 and OV-90 cells. These effects were caused by the decrease in the expression of signaling kinases involved in PI3K/MAPK cascades in ovarian cancer. Additionally, the inhibition of both cascades by inhibitors before fucoidan incubation affected the decrease in the phosphorylation of target signals and a reduction in the cell growth of ES-2 and OV-90 cells. Moreover, fucoidan triggered apoptotic proteins, induced ER stress, and decreased the expression of angiogenesis-related genes in ES-2 and OV-90 cells and exhibited synergistic effects with cisplatin or paclitaxel. Furthermore, fucoidan interrupted tumor and vascular formation in zebrafish xenograft and fli1 Tg model, respectively, whereas it did not exhibit toxicity in normal zebrafish. Thus, fucoidan can be a novel therapeutic agent through its ability to prevent the progression of ovarian cancer. However, this study also raises concerns about the effects of fucoidan in clinical application. Exposure to fucoidan in zebrafish larvae without a tumor slightly reduced larval development. This suggests that fucoidan may cause non-target effects on normal cells, not just cancer cells. Further research will increase the therapeutic efficiency of fucoidan to target specifically to cancer cells if the dose, time, and molecular targets for fucoidan-based therapy.

## Figures and Tables

**Table 1:** Information of primary antibodies.

Primary Antibodies	Dilution	Supplier	Catalog Number
Phospho-Cyclin D1 (Thr^286^)	1:1000	Cell Signaling	3300
Cyclin D1	1:1000	Cell Signaling	2922
Phospho-AKT (SER^473^)	1:1000	Cell Signaling	4060
AKT	1:1000	Cell Signaling	9272
Phospho-ERK1/2 (Thr^202^/Tyr^204^)	1:1000	Cell Signaling	9101
ERK1/2	1:1000	Cell Signaling	4695
Phospho-JNK (Thr^183^/Tyr^185^)	1:1000	Cell Signaling	4668
JNK	1:1000	Cell Signaling	9252
Phospho-P38 (Thr^180^/Tyr^182^)	1:1000	Cell Signaling	4511
P38	1:1000	Cell Signaling	9212
Phospho-P70S6K (Thr^421^/Ser^424^)	1:1000	Cell Signaling	9204
P70S6K	1:1000	Cell Signaling	9202
Phospho-S6 (Ser^235/236^)	1:1000	Cell Signaling	2211
S6	1:1000	Cell Signaling	2217
IRE1α	1:1000	Cell Signaling	3294
ATF6α	1:1000	Santa Cruz	sc-166659
Phospho-PERK (Thr^981^)	1:1000	Santa Cruz	sc-32577
PERK	1:1000	Santa Cruz	sc-13073
Phospho-eIF2α (Ser^51^)	1:1000	Cell Signaling	3398
eIF2α	1:1000	Cell Signaling	5324
GADD153	1:1000	Santa Cruz	sc-7351
GRP78	1:1000	Santa Cruz	sc-13968
VDAC	1:1000	Cell Signaling	4661
IP3R1	1:1000	Invitrogen	PA1-901
IP3R2	1:1000	Santa Cruz	sc-398434
GRP75	1:1000	Cell Signaling	3593
MFN2	1:1000	Cell Signaling	11925
VAPB	1:1000	Invitrogen	PA5-53023
LC3B	1:1000	Cell Signaling	3868
BECN1	1:1000	Cell Signaling	3495
ATG5	1:1000	Cell Signaling	12994
Cleaved caspase-3	1:1000	Cell Signaling	9664
Cleaved caspase-9	1:1000	Cell Signaling	9501
Cytochrome c	1:1000	Cell Signaling	11940
TUBA	1:2000	Santa Cruz	sc-5286
PCNA	1:100	Santa Cruz	sc-56

**Table 2:** Primer list used in quantitative RT-PCR.

Gene		Primer Sequence (5′ → 3′)		Size (bp)
Human	*VEGFA* (AF022375.1)	Forward	TTGTACAAGATCCGCAGACG	100
		Reverse	TCACATCTGCAAGTACGTTCG	
	*VEGFB* (BC008818.2)	Forward	CAGAGGAAAGTGGTGTCATGG	90
		Reverse	CATGAGCTCCACAGTCAAGG	
	*VEGFC* (NM_005429.5)	Forward	ATGTGGGGAAGGAGTTTGG	94
		Reverse	CCCTCACTATTGCAGCAACC	
	*VEGFD* (NM_004469.5)	Forward	TGTAAGTGCTTGCCAACAGC	96
		Reverse	TTTCTTGGAATGGGAACAGC	
	*FLT1* (AF063657.2)	Forward	ATGGTCTTTGCCTGAAATGG	135
		Reverse	TAGAAGCCAGTGTGGTTTGC	
	*FLT4* (AY233383.1)	Forward	GTACATGCCAACGACACAGG	131
		Reverse	TCAGGCTTGTTGATGAATGG	
	*KDR* (AF063658.1)	Forward	ACCCACGTTTTCAGAGTTGG	124
		Reverse	TCCAGAATCCTCTTCCATGC	
	*GAPDH* (BT006893.1)	Forward	GGCTCTCCAGAACATCATCC	149
		Reverse	TTTCTAGACGGCAGGTCAGG	
Zebrafish	*p53* (NM_001271820.1)	Forward	GCTTGTCACAGGGGTCATTT	94
		Reverse	ACAAAGGTCCCAGTGGAGTG	
	*casp3* (NM_131877.3)	Forward	AAAGGATCCCAGTGGAGGCAGATT	131
		Reverse	TGGTCATGATCTGCAAGAGCTCCA	
	*casp8* (NM_131510.2)	Forward	AGAGAAGGGCACAGTTTTGG	140
		Reverse	CCTGGTTCTCATCTCCTTGG	
	*casp9* (NM_001007404.2)	Forward	CTGCTGTGTGGTCATCATCC	134
		Reverse	GACAGTTCTGGCCATTGAGG	
	*vegfaa* (AF016244.1)	Forward	ATTCATACCCAGCAGCTTCG	137
		Reverse	GCAGACAGATGGAGGAGAGC	
	*vegfc* (AF466147.1)	Forward	GATGTGGGGAAAGAGTTTGG	112
		Reverse	TGATGTTCCTGCACTGAAGC	
	*flt1* (BC139515.1)	Forward	CTGGTTATTCGGGATGTTGC	121
		Reverse	TTTGGGGCTTCACATTTACC	
	*flt4* (AY833404.1)	Forward	TCACAACTGGATGGATTTGG	100
		Reverse	GCCGACAGTCTTTTCTTTGC	
	*kdr* (NM_001024653.2)	Forward	CTTGGCAGCCAGAAATATCC	116
		Reverse	GACGAGCATCTCCTTTACGG	
	*kdrl* (NM_131472.1)	Forward	CCTGATCCACAACTGCTTCC	142
		Reverse	CACACGACTCAATGCGTACC	
	*gapdh* (BC083506.1)	Forward	TGCTGGTATTGCTCTCAACG	93
		Reverse	GCCATCAGGTCACATACACG	
